# Flexible nutrient oxidation during desiccation is associated with climate stress resistance of an invasive fly

**DOI:** 10.1242/jeb.250861

**Published:** 2026-07-08

**Authors:** Henrika J. Bosua, Marshall D. McCue, Christopher W. Weldon, John S. Terblanche

**Affiliations:** ^1^ Centre for Invasion Biology, Department of Conservation Ecology and Entomology, Stellenbosch University, Stellenbosch 7600, South Africa; ^2^ Department of Zoology, Stockholm University, SE-106 91 Stockholm, Sweden; ^3^ Sable Systems International, North Las Vegas, NV 89032, USA; ^4^ Department of Zoology and Entomology, University of Pretoria, Hatfield 0028, South Africa

**Keywords:** Population dynamics, Environmental physiology, Metabolic pathways, Nutrient oxidation, Tephritidae, Desiccation, Stable isotopes, Insects, Respirometry, Trait–environment relationships

## Abstract

The capacity of living organisms to withstand stress may be energetically expensive, thereby placing a demand on body nutrient stores and any nutrients that might be linked to coping with that stress. Here, we tested the hypothesis that desiccation-resistant *Ceratitis capitata* (Diptera: Tephritidae) and desiccation-sensitive *Ceratitis rosa* differentially oxidise nutrients to counter desiccation stress and facilitate recovery. Lines of flies from both species had their body macronutrient stores experimentally enriched with one of three ^13^C tracers: leucine (enriches proteins), palmitic acid (enriches lipids) and glucose (enriches primarily carbohydrates), and were then subjected to a regimen of desiccation stress followed by recovery. We used flow-through respirometry and ^13^C-breath testing to detect changes in metabolic rates and macronutrient oxidation. *Ceratitis capitata*, unlike *C. rosa*, showed highly flexible metabolic rates and nutrient oxidation. The former upregulated oxidation of both carbohydrates and lipids whereas protein oxidation was only upregulated during recovery from desiccation. This provides novel support for biochemical pathway (macronutrient) flexibility enhancing the survival of a desiccation-resistant invasive species over a desiccation-sensitive species.

## Teaser

**Summary:** Selective nutrient oxidation enhances desiccation resistance in an invasive insect pest species.

## INTRODUCTION

Environmental conditions affect nutrient assimilation at all trophic levels. Temperature and water availability influence the amount and ratio of foliage nutrients, and plants from arid environments have less carbon and nitrogen stored in foliage than those from warmer and wetter climates (Sardans et al., 2020). The temperature at which an insect feeds and grows will also affect how nutrients are assimilated into body stores; for example, mealworms (*Tenebrio molitor*) reared at temperatures below 20°C and higher than 30°C had lower protein stores but higher lipid stores than individuals reared at a benign (25°C) temperature (Bjørge et al., 2018). In some cases, insects reared at lower temperatures have been found to have both slower growth and greater nutrient reserves than individuals of the same species reared at higher temperatures (Kukal and Dawson, 1989; Coggan et al., 2011). Changing global climates will likely affect nutrient accumulation in herbivorous insects both indirectly through changes in plant nutrient stores and directly by affecting their ability to digest and assimilate these nutrients (Hamann et al., 2021). Indeed, metabolic flexibility of insects may be key to insect persistence with climate change and overcoming diverse anthropogenic stressors (Terblanche and Lehmann, 2026).

Nutrients consumed during juvenile stages and accumulated in the body stores of adults can be mobilised as needed for adult survival and reproduction (Andersen et al., 2010; Briegel, 1990; Carey et al., 2005; Chen et al., 2017; Municio et al., 1971; Merli et al., 2018; Levin et al., 2017a). For some insects, these body stores can be more important than the nutrients acquired during adult feeding. In migratory locusts, daily refuelling remains insufficient even when energy-rich food is abundantly available, owing to limitations to intake and assimilation; consequently, these insects rely on stored carbohydrates and lipids to sustain daily flight (Cease et al., 2026). Adult fruit flies, when faced with stressful conditions, mobilise body stores accumulated through the larval diet to offset this stress rather than relying on compensatory feeding (Weldon et al., 2019). Furthermore, specific nutrients can be preferentially oxidised during certain environmental stressors (Levin et al., 2017b; McCue et al., 2015). For example, carbohydrate-biased diets increase both desiccation and starvation resistance in mosquitoes but prolong developmental time (Kristensen et al., 2016; Pascacio-Villafán et al., 2016). During times of desiccation stress, metabolic water is an important avenue of water gain for several species, both through the digestion of ingested nutrients and also by oxidising body nutrients stored previously. The nutrient catabolised during times of water stress varies with species and environmental conditions; for example, protein is catabolised by migrating birds to combat water loss and provide fuel for flight (Gerson and Guglielmo, 2011), while resting zebra finches without access to food or water catabolise more fat stores than fasting finches with access to water (Rutkowska et al., 2016). Whole-body lipolysis increased in dehydrated human males (Keller et al., 2003), while dehydrated scorpions adapted to arid conditions catabolise more stored carbohydrates, and switch to a mixture of metabolic fuels when not stressed (Kalra and Gefen, 2012). Because the oxidation of stored lipids releases twice as much metabolic water as the oxidation of protein and carbohydrates, it may be expected that insect lipid stores will be preferentially metabolised at greater rates in times of desiccation stress compared with other body stores (Weldon et al., 2019; Heath et al., 1971; Nagy, 1976; Calder and Braun, 1983).

Stable isotopes of molecules (such as ^13^C or ^15^N) commonly occurring in organic compounds can be used to trace nutrients through several trophic levels, and doubly labelled water (DLW) can trace movements of water through animals and ecosystems (McCue et al., 2020). How efficiently dietary nutrients are incorporated into body tissues and which body stores are mobilised under diverse conditions or through time can be determined by artificially enriching larval diet with a ^13^C tracer that becomes incorporated into specific macronutrient pools. As these labelled body stores are oxidised, the CO_2_ produced will contain ^13^C as the enriched molecule is cleaved off during specific biochemical reactions. Thus, this technique can be used to trace, in a safe, non-destructive manner, nutrient usage under a range of experimental manipulations (McCue et al., 2016, 2011, 2010; Welch et al., 2016; McCue and Welch, 2016) and thus probe key questions in macronutrient utilisation during stress.

**Fig. 1.**
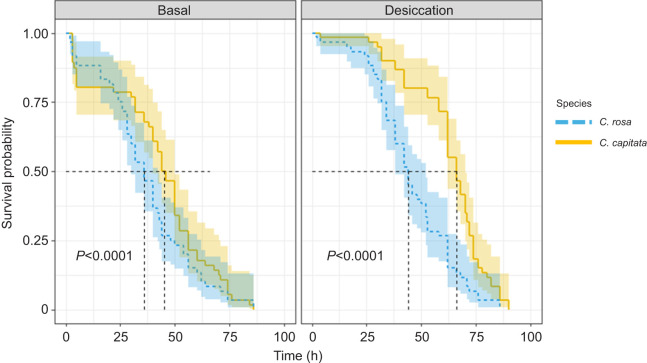
**Survival of *Ceratitis capitata* and *Ceratitis rosa* under desiccation conditions.** Responses of non-acclimated individuals under basal (left) and desiccation (25°C and 0% relative humidity, RH; right) conditions are shown. *P*-values indicate the 50% survival probability (Cox proportional hazard test).

Desiccation-resistant species are perhaps more likely to switch to using lipids in dry conditions than less desiccation-tolerant species. A comparison of fruit fly species under dry conditions showed that lipid reserves of a desiccation-tolerant species (*Ceratitis capitata*) were more depleted after a desiccation event than can be explained by starvation alone, and the degree of utilised lipid reserves was proportionally more than that of the desiccation-sensitive species to which it was compared (Weldon et al., 2016). As a highly invasive species found in a range of climates, *C. capitata* serves as a good model species to determine various environmental adaptations that might improve survival. Recent studies show that *C. capitata* is more desiccation tolerant than other closely related species (Bosua et al., 2022; Weldon et al., 2016; Fig. 1) and that metabolic water gain might contribute to this tolerance (Weldon et al., 2016). Therefore, the role that flexible nutrient oxidation plays in withstanding desiccation stress through the production of metabolic water can be investigated by comparing these model fruit fly species.

In this study, we investigated whether the ability to differentially oxidise nutrients to counter desiccation stress and facilitate recovery varied between two insect pest species with different levels of desiccation resistance. The macronutrient pools in the body stores of adults were traced throughout different environmental conditions by rearing larvae on diets enriched with specific ^13^C stable isotope tracers (lipids, proteins or carbohydrates) to estimate reliance on specific fuels under desiccation stress and during recovery from desiccation. We predicted that during desiccating conditions, the desiccation-tolerant species, *C. capitata*, would utilise lipid stores better (i.e. metabolise lipids faster and/or to a greater extent), thereby producing more metabolic water. In contrast, the less desiccation-tolerant species, *C. rosa*, would exhibit this selective metabolism of lipids to a lesser extent. Furthermore, we predicted that desiccation-tolerant species might be able to adaptively reduce *V̇*_CO_2__, to conserve water loss rates.

## MATERIALS AND METHODS

### Fly collection, handling and rearing

*Ceratitis capitata* (Wiedemann 1824) was selected as a desiccation-tolerant species and *Ceratitis rosa* Karsch 1887 was selected to represent a desiccation-sensitive species (based on the results of Weldon et al., 2016; Bosua et al., 2022). Mean body size did not differ significantly between the species, but females were significantly larger than males in both (Fig. S1). *Ceratitis capitata* and *C. rosa* were collected from infested fruit in the Stellenbosch and Nelspruit areas of South Africa, respectively, and used to establish laboratory cultures using standard methods (see Supplementary Materials and Methods, ‘S1 Fly rearing and handling’). All measurements were performed on laboratory-reared, F_2_ generation male and female fruit flies. All food and water were removed 2 h prior to metabolic and nutritional measures to ensure that the only source of nutrients for the flies would be derived from stored body tissues.

### Stable isotope (^13^C) tracer molecules

#### Tracer molecules and diet preparation

For both species, the diets of three parallel groups of larvae reared under otherwise identical conditions (see Supplementary Materials and Methods, ‘S1 Fly rearing and handling’) were enriched with U-^13^C_6_-glucose (Glu), 1-^13^C palmitic acid (Palm) or 1-^13^C-leucine (Leu) (Cambridge Isotope Laboratories, Tewksbury, MA, USA) at a concentration of 2.0 g l^−1^. Assuming that all were equally digested, assimilated and mobilised from the flies' body, the ^13^C labels from each ingested nutrient would be incorporated into body tissues. Labelled glucose, palmitic acid or leucine result in more ^13^C in carbohydrate, lipid or protein body stores, respectively. By sampling exhaled breath from flies of each species fed different ^13^C-labelled larval diets, we were then able to determine which body store was oxidised during desiccation stress. If ^13^C was more prominent in the gas samples from one of the groups with enriched Glu, Palm or Leu, we could then infer that the targeted nutrient was being preferentially metabolised.

#### Sampling intervals

**Fig. 2.**
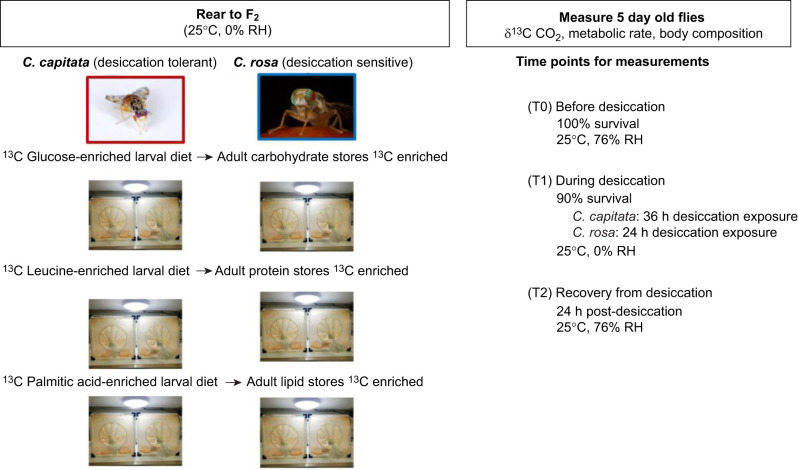
Outline of the experimental treatments and measurement timeline for desiccation-tolerant *C. capitata* and desiccation-sensitive *C. rosa*.

Measurements were made on 5 day old flies reared under benign conditions (25°C, 76% relative humidity, RH). Following the experimental design of Williams et al. (2016), three sampling intervals were selected: 100% survival (Before; no desiccation stress), immediately following desiccation pre-treatment for 90% survival (During) and after desiccation pre-treatment until 90% survival followed by recovery for 24 h without food and water (Recovery) (Fig. 2). The time in hours that 90% of *C. capitata* and *C. rosa* were predicted to withstand desiccation for was taken from previously published estimates (Bosua et al., 2022; Weldon et al., 2016). From these data, the 90% survival time for *C. capitata* was taken as 36 h and that for *C. rosa* as 24 h, while the 50% survival time was taken as 48 h for *C. capitata* and as 33 h for *C. rosa*.

### Respirometry and breath samples

Concentrations of labelled ^13^C tracers (molar flux rates) oxidised during desiccation stress were calculated in both fly species with repeated alternating measures of gas ‘breath’ samples and respirometry trials. The protocols were adapted from the tsetse fly experiments of McCue et al. (2016) and adjusted to the mass and metabolic rate of individual tephritid flies, as adapted from the Williams et al. (2016) *Drosophila*-based protocols.

#### Respirometry

Respirometry equipment was set up as described in Supplementary Materials and Methods, ‘S1 Fly rearing and handling’. To determine how metabolic rate and nutrient utilisation change over a period of desiccation stress in two species of flies with different desiccation physiology, individuals from each species and treatment group were subjected to an alternating regimen of metabolic rate measurement followed by a rest (incubation) period. Respirometry measurements were taken under desiccating conditions (25°C and 0% RH) in CO_2_-free air with flow-through respirometry systems (LiCor 7000 CO_2_/H_2_O gas analyser) at a flow rate of 100 ml h^−1^, using similar methods to those in Bosua et al. (2022). Metabolic rates were recorded as the rate of carbon dioxide production (*V̇*_CO_2__; ml h^−1^) using flow-through respirometry.

For each of the three sampling intervals, ten 5 day old flies of each sex were randomly selected from each species and treatment and weighed individually to the nearest 0.1 mg (Mettler Toledo, MS104S/01). Metabolic measurements were recorded from groups of females and males (*n*=7 each) at 25°C in separate 5 ml chambers with a push mode 8-channel multiplexing respiratory system (RM8 Intelligent Multiplexer, V5, Sable Systems International). Each of the seven fly-containing channels was measured for 15 min per channel, and the remaining empty channel recorded a baseline CO_2_ reading, later used to drift-correct metabolic measurements of flies if necessary. The remaining three individuals were weighed along with the rest of the flies and their mass-specific metabolic rates were estimated based on the scaling relationships determined from the seven flies tested and existing data for the species (as in McCue et al., 2016).

An electronic activity detector (Sable Systems International AD-1) was connected to at least one of the respirometry chambers per multiplexer run, to qualitatively identify resting or active periods. To keep the flies quiescent in the respiratory chamber, natural light was excluded by wrapping the system in aluminium foil and closing the bath lid. Gas concentrations were recorded on LiCor software and Sable Systems International software (Expedata) was used to extract and calculate *V̇*_CO_2__ using standard equations as per Williams et al. (2016) and Bosua et al. (2023).

#### Stable isotope gas ‘breath’ samples

Immediately following the 2 h respirometry trial, the groups of flies were sealed in 20 ml respiratory chambers which were then sealed with a luer cap, thus sealing in the dried air and continuing the desiccation exposure treatments. The respiratory chambers were then placed in an incubator under standard conditions (25°C) for 16 h to allow CO_2_ concentrations to accumulate to a detectable amount (i.e. >2%), after which the breath sample was collected. The duration (h) required for flies to produce this amount of CO_2_ depends on *V̇*_CO_2__. Basson et al. (2012) reported the *V̇*_CO_2__ of *C. capitata* to be between 0.010 and 0.014 ml h^−1^, while Bosua et al. (2023) found a *V̇*_CO_2__ of 0.084 ml h^−1^ for the same species. Using these values, we estimated that an individual fly would take between 5 h (using values from pilot trails) and 33 h (using values from Basson et al., 2012) to produce sufficient CO_2_. Because three bouts of breath samples were collected to measure changes in nutrient metabolism over time, the total time to collect these samples was between 15 and 99 h. However, both *C. capitata* and *C. rosa* have average survival times of <50 h when exposed to desiccating conditions (Weldon et al., 2016); therefore, a breath sampling collection time of 16 h was used, as this falls between the two values calculated for sufficient CO_2_ production while also permitting fly survival for the full duration of the experiment.

Breath samples of individual flies were collected by ejecting 15 ml of the gas from each individual plastic syringe into evacuated Exetainer vials (Labco Limited, Lampeter, UK). After a breath sample was taken, the respiratory chambers were flushed with room air with standard atmospheric concentrations of CO_2_ (i.e. 0.04%) and reconnected to the multiplexer.

The amounts of labelled ^13^C (δ^13^C values in terms of international VPDB standards) in each breath sample were measured with a Helifan Plus (Fischer Analysen Instrumente, Leipzig, Germany) non-dispersive infrared spectrometer interfaced with a FanAS auto sampler (as described by Williams et al., 2016). The spectrometer was calibrated by running vials containing CO_2_ with known δ^13^C before and after each batch of unknown samples.

Three rounds of respirometry [at sampling intervals before, during and after (recovery) desiccation, as detailed above; Fig. 2], each followed by a 16 h rest period were conducted to gain an overview of which nutrients were utilised by the flies during desiccation. After the final round of respirometry, the flies were flash-frozen in liquid nitrogen and stored at −80°C for later biochemical analysis. Macronutrient composition (body water, lipid, protein and carbohydrate stores) was determined in separate groups of flies for each species, tracer and time point to validate that similar results were obtained when comparing these two species with work reported previously (Weldon et al., 2016; detailed methods are given in Supplementary Materials and Methods, ‘S3 Body composition’).

### Data analysis

Lower metabolic rates are linked to reduced respiratory water loss. Therefore, we compared the mass-specific *V̇*_CO_2__ (ml h^−1^ mg^−1^) between *C. capitata* and *C. rosa* at different time points and tracer enrichment treatments with generalised linear models (GLMs), followed by pairwise slope comparisons. We tested whether metabolic rate differed between time points in *C. rosa* and *C. capitata*. Given our prediction of greater metabolic flexibility in the more desiccation-tolerant *C. capitata* compared with the less desiccation-tolerant *C. rosa*, we expected the slopes of metabolic rate change to be greater in *C. capitata* than in *C. rosa*. Considering that the two species have very similar body mass (*C. capitata*: 10.16±1.17 mg and *C. rosa*: 10.27±0.99 mg; see Fig. S1), mass-specific metabolic rates can be compared with greater confidence (Raubenheimer, 1995).

We expected metabolism of specific macronutrients during desiccation stress would differ between the two species; therefore, the amount of ^13^C tracers detected in the gas samples was predicted to differ between *C. capitata* and *C. rosa*. To test this prediction, we compared the ^13^C tracers in gas (breath samples) between the species and time points within each tracer using GLMs, with body mass added as a covariate. Where the two species differed in nutrient tracer oxidation over time (significant species×time point interaction), the slope of the change between the paired time points (initial to desiccation, desiccation to recovery, and initial and recovery) was compared with pairwise slope contrasts. Furthermore, to determine whether either one of the two species displayed more flexibility in mobilisation of a specific tracer, the effect sizes for each response (metabolic rate and nutrient oxidation rate) were determined with Cohen's *d* test and compared between species for each time point and tracer, and body mass was added as a covariate. Should *C. capitata* be more flexible in mobilising a specific tracer, we would expect to find a larger standardised effect size than in *C. rosa*, as reflected by a higher Cohen's *d*. Cohen's *d* values of 0.2, 0.5 and 0.8 or above indicate small, medium and large effect sizes, respectively.

The ^13^C gas breath samples collected from tracer-enriched flies were reported as the amounts relative to unenriched samples, and therefore the atom fraction excess (AFE) was modelled for each species, treatment and sex from the equation of McCue et al. (2016). In this equation, the VPDB is a constant (IAEA, 2000). At each time point, the instantaneous rate of tracer oxidation (*T*) for each of the species, sex and enrichment treatment was calculated as the product of the *V̇*_CO_2__ of each fly and the related AFE (for that specific sample), divided by the molar mass of each tracer (*m*) and volume of CO_2_ produced per gram of mixed substrate oxidised using a value of 1.01 g^−1^ (*K*) (McCue et al., 2016): *T*=(*V̇*_CO_2__×AFE)/*mK*.

If the metabolism of specific nutrients such as lipids is linked to the desiccation physiology of fruit flies, we would expect that the flies would either store different amounts of these nutrients or metabolise them at different rates. The amounts of protein, lipid and carbohydrate present before, during and after recovery from desiccation stress were compared between *C. capitata* and *C. rosa* with GLMs, and differences between the groups were determined with pairwise slope contrasts.

Statistical analyses were conducted in R (version 4.3.1; https://www.r-project.org/). GLMs were fitted using the stats package (version 4.3.1). Estimated marginal means and pairwise slope contrasts were computed using the emmeans package (version 1.8.7). Data manipulation and visualisation were performed using dplyr and ggplot2, and expressed as means±s.e.m. unless otherwise specified.

## RESULTS

### Metabolic rate

**Fig. 3.**
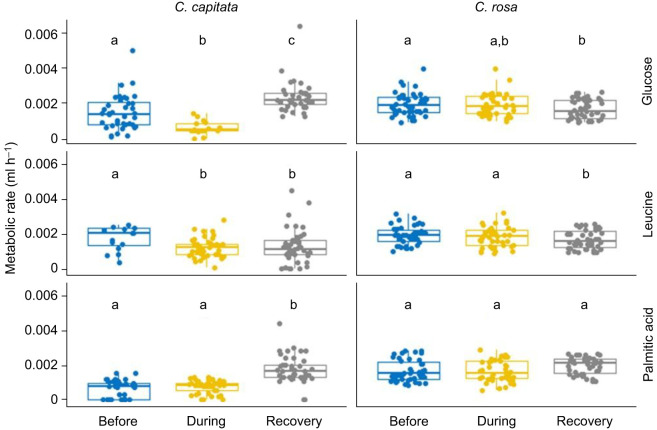
**Individual metabolic rates for each species for each of the three metabolic tracers tested (glucose, leucine and palmitic acid).** Metabolic rate was calculated for *C. capitata* and *C. rosa* from ^13^C gas samples. The three time points represented are: before – benign conditions of 76% RH, 25°C; during – desiccation conditions of <2% RH, 25°C; and recovery – benign conditions of 76% RH, 25°C after the desiccation event. Box plots show medians, upper and lower quartiles and 1.5× the interquartile range, with individual data points. Significant differences in metabolic rate between time points are indicated by different letters for each nutrient and species, based on pairwise comparisons of estimated marginal means from GLMs using emmeans with Tukey-adjusted *P*-values.

Piecewise linear analyses (GLMs) of *V̇*_CO_2__ revealed substrate-specific temporal differences in metabolic dynamics between *C. capitata* and *C. rosa*. For glucose-associated metabolism, *C. capitata* showed a significant decline in *V̇*_CO_2__ during the initial interval [before→during: slope=−8.72±2.28(×10^−4^); Fig. 3], whereas *C. rosa* showed no significant change [slope=3.94±1.68(×10^−4^); Fig. 3], producing a significant species difference (estimate=−9.11×10^−4^, *P*=0.0015; Fig. 3). During the later interval (during→recovery), *C. capitata* exhibited a strong increase in *V̇*_CO_2__ [slope=18.09±2.31(×10^−4^); Fig. 3], while *C. rosa* showed a non-significant decline [slope=−3.08±1.68(×10^−4^); Fig. 3], with a highly significant interspecific contrast (estimate=21.2×10^−4^, *P*<0.0001). Correspondingly, *C. capitata* showed a significant shift in slope after desiccation stress (*F*=40.06, *P*<0.001), whereas *C. rosa* did not (*P*=0.234), and the magnitude of the slope change differed significantly between species (*F*=34.72, *P*<0.001), yielding a strong species×time interaction (*F*=29.20, *P*<0.001). Effect sizes of *V̇*_CO_2__ between the species ranged from moderate in the initial condition (*d*=−0.573) to very large during desiccation stress (*d*=−2.27), indicating higher metabolic rate in *C. rosa* during stress, followed by a large positive effect during recovery (*d*=0.943), suggesting more flexible metabolic rates in *C. capitata*.

For leucine-associated metabolism, *C. capitata* again showed an early decline in *V̇*_CO_2__ [slope=−8.75±2.21(×10^−4^); Fig. 3], whereas *C. rosa* showed no significant change [slope=−0.343±1.64(×10^−4^); Fig. 3], resulting in a significant species difference (*P*=0.0025). No significant trends occurred in the later interval for either species, nor was the interspecific contrast significant (*P*=0.249). Nevertheless, *C. rosa* showed a significant change in slope after desiccation (*F*=8.54, *P*=0.0038), unlike *C. capitata* (*P*=0.544), with a significant difference in slope change between species (*F*=6.71, *P*=0.010) and a significant species×time interaction (*F*=4.68, *P*=0.010). Effect sizes for leucine were small to moderate (initial: *d*=0.189; desiccation stress: *d*=−0.990; recovery: *d*=−0.482), indicating generally modest interspecific differences.

For palmitic acid-associated metabolism, temporal patterns also differed between species but were primarily expressed during recovery. In the initial interval, *V̇*_CO_2__ trends were similar between *C. capitata* [slope=1.17±1.35(×10^−4^)] and *C. rosa* [slope=0.494±1.32(×10^−4^)], with no significant species difference (estimate=0.681×10^−4^, *P*=0.719). However, during the later interval, *C. capitata* exhibited a significantly steeper increase in *V̇*_CO_2__ [slope=10.11±1.33(×10^−4^)] than *C. rosa* [slope=2.44±1.32(×10^−4^)], producing a significant interspecific contrast (estimate=7.68×10^−4^, *P*<0.001). Within-species tests confirmed a significant post-desiccation slope change in *C. capitata* (*F*=14.84, *P*<0.001) but not in *C. rosa* (*P*=0.398), and the magnitude of the slope change differed significantly between species (*F*=4.60, *P*=0.033), resulting in a strong species×time interaction (*F*=12.22, *P*<0.001). Effect sizes were large and negative at the initial condition (*d*=−1.88) and desiccation stress (*d*=−1.59), but small at recovery (*d*=−0.311), indicating higher lipid-supported metabolic rates in *C. rosa*, particularly at earlier time points. Overall, these findings reveal substrate-dependent differences in metabolic physiology between the species. The most pronounced divergence occurred in lipid metabolism, whereas carbohydrate and amino acid metabolism showed comparatively modest or transient differences. These results identify lipid utilisation as a key physiological distinction between the two species during and after desiccation stress.

### Nutrient oxidation

**Fig. 4.**
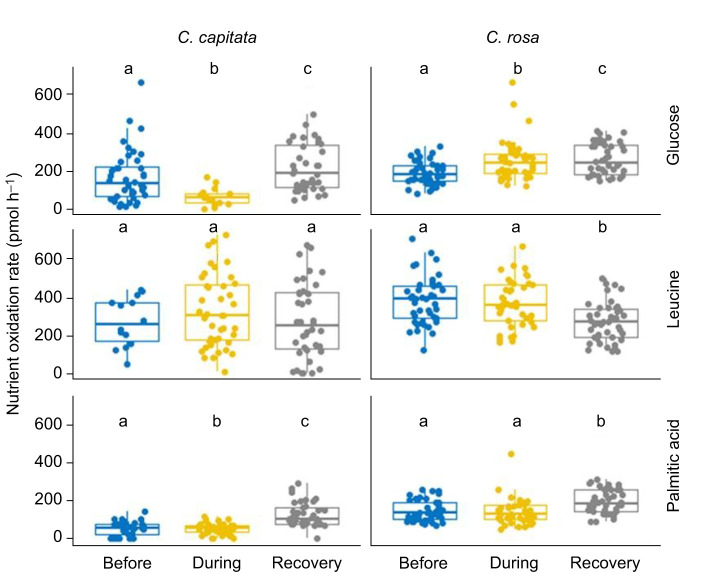
**Nutrient oxidation rates for each species for each of the three metabolic tracers tested (glucose, leucine and palmitic acid).** Nutrient oxidation rate was calculated for *C. capitata* and *C. rosa* from ^13^C gas samples, before, during and after (recovery) the desiccation event (see Fig. 3). Significant differences in nutrient oxidation rates between time points are indicated by different letters for each nutrient and species, based on pairwise comparisons of estimated marginal means from GLMs using emmeans with Tukey-adjusted *P*-values.

Piecewise analyses (GLM within each tracer) revealed distinct species-specific patterns of nutrient oxidation. For glucose, *C. capitata* showed a significant decline during desiccation (slope=−102.3±35.1; Fig. 4) followed by a strong increase during recovery (slope=176.49±35.7; Fig. 4), whereas *C. rosa* showed an early increase during desiccation (slope=67.6±25.9; Fig. 4) and another, smaller but significant, increase during recovery (slope=5.28±25.9; Fig. 4), producing significant species differences in both intervals (estimate=−170 and 171, *P*<0.001) and a significant species×time interaction (*F*=9.25, *P*<0.001). A significant slope change occurred within *C. capitata* (*F*=18.18, *P*<0.001) but not *C. rosa* (*P*=0.166), and slope change magnitude differed between species (*F*=18.50, *P*<0.001). Effect sizes were small at initial and recovery stages (before, *d*=−0.201; recovery, *d*=−0.142) but large during desiccation (*d*=−2.32), indicating substantially higher glucose oxidation in *C. rosa* under stress.

For leucine, early responses did not differ between species (*C. capitata*: slope=−11.82±65.5; *C. rosa*: slope=−1.22±48.6; estimate=−10.6, *P*=0.897), but during recovery, *C. capitata* showed a positive trend (slope=50.8±48.6) while *C. rosa* declined significantly (slope=−111.4±48.6), resulting in a significant species difference (estimate=162, *P*=0.019) and species×time interaction (*F*=3.24, *P*=0.041). Within-species slope changes were not significant (*C. capitata*: *P*=0.510; *C. rosa*: *P*=0.192), and effect sizes remained small across time points (*d*=−0.235 to 0.368), suggesting limited biological differences in amino acid utilisation.

Palmitic acid oxidation showed broadly similar temporal patterns in both species. During the initial interval, *C. capitata* lipid oxidation showed a small, positive trend (slope=5.25±12.5), while *C. rosa* (slope=−1.46±12.3) showed no significant changes, but species differences were not significant (estimate=6.7, *P*=0.702). During recovery, oxidation increased in both species (*C. capitata*: slope=72.9±12.3; *C. rosa*: slope=51.4±12.3), and the species×time interaction was significant (*F*=1.43, *P*=0.0240). Both species showed significant within-species slope increases (*C. capitata*: *F*=9.91, *P*<0.001; *C. rosa*: *F*=6.19, *P*=0.0135) and the slope of the change in palmitate oxidation between the stress period and recovery period was greater in *C. capitata* than in *C. rosa*. The effect size between the species was consistently large at each time point (before, during and recovery, *d*=2.05, 1.74, 1.08, respectively), indicating fatty acid oxidation is more flexible in *C. capitata*.

Overall, carbohydrate metabolism was species and time point specific. Amino acid utilisation differed modestly, and lipid metabolism consistently indicated greater reliance on fatty acid substrates during and after desiccation in *C. capitata* relative to *C. rosa*.

### Body composition

Desiccation induced significant changes in body composition and metabolite pools in both *C. capitata* and *C. rosa*, with distinct species-specific responses depending on tracer type.

#### Body water

Body water showed species-specific temporal responses to desiccation. A significant species×time interaction was observed in glucose-enriched flies (*F*=6.49, *P*=0.012) and leucine-enriched flies (*F*=6.76, *P*=0.011), but not in palmitic acid-enriched flies (*F*=2.73, *P*=0.092). Overall, *C. capitata* exhibited greater short-term fluctuations in hydration, with body water typically declining during exposure (∼1.3–1.9 mg) and stabilising during recovery to near or below pre-exposure levels. In contrast, *C. rosa* showed smaller decreases during exposure and continued reductions during recovery, while consistently maintaining higher absolute body water than *C. capitata* (generally ∼1.5–2.3 mg greater). These patterns suggest greater temporal variability in hydration in *C. capitata*, whereas *C. rosa* maintained higher baseline water content despite progressive depletion. Species-level patterns reflected these broader trends. In *C. capitata*, body water declined from pre- to post-desiccation stages across all tracers (e.g. glucose tracer: 5.30±0.87→4.93±0.29 mg; leucine: 4.92±0.77→4.50±1.32 mg; palmitic acid: 5.05±0.37→4.73±0.42 mg; *P*<0.05, Table 1). Similarly, *C. rosa* showed moderate reductions in body water during desiccation, although water proportions remained consistently higher than in *C. capitata* across all time points.

**Table 1.** Body stores of carbohydrate, protein and lipid for desiccation-tolerant *Ceratitis capitata* and desiccation-sensitive *Ceratitis rosa* before, during and after (recovery) a desiccation event

Tracer	Body store	Before		During		Recovery	
		Mass (mg)	Proportion	Mass (mg)	Proportion	Mass (mg)	Proportion
*C. capitata*							
Glucose	Wet mass	9.28±0.92		9.28±0.34		8.08±0.25	
	Body water	5.27±0.87	0.71±0.03^a^	6.60±0.23	0.57±0.08^b^	4.93±0.29	0.61±0.03^c^
	Dry mass	4.00±0.90	0.29±0.03^a^	2.68±0.32	0.43±0.08^b^	3.09±0.27	0.38±0.02^a^
	Carbohydrate	0.28±0.05	0.04±0.01^a^	0.10±0.02	0.07±0.01^b^	0.15±0.01	0.05±0.01^c^
	Lipid	0.23±0.03	0.06±0.01^a^	0.17±0.02	0.06±0.01^b^	0.21±0.02	0.07±0.01^a^
	Protein	0.63±0.12	0.26±0.03^a^	0.68±0.05	0.16±0.04^a^	0.65±0.05	0.21±0.03^b^
Leucine	Wet mass	8.46±0.54		8.74±1.11		7.33±1.05	
	Body water	4.92±0.77	0.78±0.05^a^	6.84±1.16	0.58±0.06^b^	4.50±1.32	0.61±0.09^c^
	Dry mass	3.55±0.33	0.23±0.04^a^	1.96±0.22	0.42±0.06^b^	2.83±0.46	0.39±0.09^c^
	Carbohydrate	0.57±0.19	0.04±0.01^a^	0.08±0.01	0.16±0.05^b^	0.13±0.02	0.05±0.01^b^
	Lipid	0.27±0.03	0.07±0.01^a^	0.13±0.02	0.08±0.01^b^	0.21±0.02	0.08±0.01^a^
	Protein	0.81±0.09	0.24±0.05^a^	0.45±0.04	0.23±0.03^b^	0.99±0.16	0.36±0.09^a^
Palmitic acid	Wet mass	8.87±0.56		9.01±0.80		7.70±1.05	
	Body water	5.05±0.37	0.71±0.05^a^	6.41±0.61	0.57±0.02^b^	4.73±0.42	0.62±0.07^c^
	Dry mass	3.82±0.31	0.29±0.05^a^	2.60±0.61	0.43±0.02^b^	2.97±0.93	0.38±0.07^a^
	Carbohydrate	0.23±0.02	0.04±0.02^a^	0.10±0.03	0.06±0.01^b^	0.14±0.02	0.05±0.01^c^
	Lipid	0.26±0.01	0.07±0.02^a^	0.17±0.03	0.07±0.01^b^	0.20±0.06	0.07±0.01^a^
	Protein	0.26±0.05	0.07±0.02^a^	0.17±0.04	0.07±0.01^b^	0.20±0.01	0.07±0.02^a^
*C. rosa*							
Glucose	Wet mass	11.56±0.54		10.17±1.92		9.61±1.61	
	Body water	7.44±0.25	0.72±0.05^a^	7.36±1.56	0.64±0.04^b^	6.40±1.04	0.67±0.02^a^
	Dry mass	4.13±0.64	0.28±0.05^a^	2.82±0.69	0.36±0.04^b^	3.21±0.64	0.33±0.02^c^
	Carbohydrate	0.40±0.02	0.06±0.02^a^	0.17±0.06	0.10±0.01^b^	0.21±0.06	0.06±0.02^b^
	Lipid	0.26±0.02	0.06±0.02^a^	0.16±0.07	0.06±0.01^a^	0.25±0.06	0.08±0.02^b^
	Protein	1.37±0.19	0.31±0.07^a^	0.84±0.12	0.33±0.03^b^	0.66±0.10	0.21±0.03^c^
Leucine	Wet mass	10.83±1.07		9.35±1.51		9.79±1.36	
	Body water	6.81±1.09	0.69±0.07^a^	6.45±0.94	0.63±0.06^b^	5.19±0.84	0.53±0.05^c^
	Dry mass	4.01±0.51	0.31±0.07^a^	2.91±0.93	0.37±0.06^b^	4.60±0.78	0.47±0.05^c^
	Carbohydrate	0.43±0.06	0.07±0.03^a^	0.18±0.06	0.11±0.03^b^	0.09±0.02	0.02±0.01^c^
	Lipid	0.22±0.08	0.06±0.02^a^	0.16±0.03	0.05±0.01^a^	0.25±0.15	0.06±0.04^b^
	Protein	1.00±0.26	0.39±0.14^a^	1.04±0.19	0.26±0.09^b^	1.37±0.39	0.31±0.11^c^
Palmitic acid	Wet mass	11.21±1.35		9.49±0.74		10.00±0.83	
	Body water	7.17±1.21	0.73±0.06^a^	6.88±0.32	0.64±0.05^b^	6.67±0.86	0.67±0.06^b^
	Dry mass	4.04±0.49	0.27±0.06^a^	2.61±0.77	0.36±0.05^b^	3.33±0.70	0.33±0.06^a^
	Carbohydrate	0.38±0.10	0.06±0.01^a^	0.16±0.05	0.10±0.03^b^	0.21±0.03	0.07±0.02^a^
	Lipid	0.25±0.05	0.06±0.02^a^	0.14±0.03	0.06±0.02^a^	0.25±0.08	0.08±0.03^b^
	Protein	1.35±0.39	0.31±0.09^a^	0.77±0.10	0.34±0.10^b^	1.72±0.47	0.55±0.22^c^

Data are absolute values (±s.d.; *n*=6 per tracer and time point) and proportion contribution to total wet mass of body water as well as dry mass body stores of carbohydrate, lipids and protein for the three tracers (^13^C-glucose, ^13^C-leucine and ^13^C-palmitic acid) for each of the three time points: before – benign conditions of 76% relative humidity (RH), 25°C; during – during desiccation conditions of <2% RH, 25°C; and recovery – recovery in benign conditions of 76% RH, 25°C, after the desiccation event. Differences in proportions before, during and after desiccation were determined with generalised linear models (GLMs) for each species and tracer (indicated with different letters).

#### Carbohydrates

Carbohydrate responses varied among tracers. The species×time interaction was not significant in glucose-enriched flies (*F*=2.08, *P*=0.168) but was significant in leucine-enriched (*F*=55.64, *P*<0.001) and palmitic acid-enriched flies (*F*=13.06, *P*<0.001). In *C. capitata*, carbohydrate content showed the strongest depletion during desiccation (glucose tracer: 0.28±0.01→0.10±0.03 mg), followed by partial restoration during the post-desiccation phase (Table 1). A similar pattern occurred in *C. rosa*, where carbohydrate pools declined significantly during desiccation (glucose tracer: 4.13±0.28→2.81±0.36 mg; carbohydrate: 0.40±0.02→0.17±0.03 mg; *P*<0.05, Table 1), followed by substantial recovery after rehydration (0.10±0.03→0.15±0.02 mg; *P*<0.05, Table 1).

#### Proteins

Protein responses differed between species and tracers. The species×time interaction was significant in glucose-enriched (*F*=0.49, *P*=0.006) and leucine-enriched flies (*F*=1.55, *P*=0.025), but not in palmitic acid-enriched flies (*F*=15.39, *P*=0.127). In *C. capitata*, protein levels remained comparatively stable overall, except under the leucine tracer, where protein content decreased significantly during desiccation (0.81±0.05→0.45±0.10 mg; *P*<0.05) before recovering post-stress (Table 1). By contrast, *C. rosa* showed an opposing pattern, with protein mass increasing sharply following desiccation across all tracers (e.g. glucose tracer: 0.83±0.12→1.64±0.17 mg; *P*<0.05).

#### Lipids

Lipid responses also differed between species and tracer treatments. The species×time interaction was not significant in glucose-enriched flies (*F*=0.96, *P*=0.411) but was significant in leucine-enriched (*F*=23.51, *P*=0.007) and palmitic acid-enriched flies (*F*=16.47, *P*<0.001; Table 1). In *C. capitata*, lipid stores declined consistently during desiccation. In contrast, *C. rosa* showed only minor fluctuations in lipid content, indicating a comparatively more stable lipid reserve during water stress.

## DISCUSSION

As predicted, the desiccation-resistant species, *C. capitata*, showed more flexibility than *C. rosa* in terms of both metabolic rates and nutrient oxidation. Notably, *C. capitata* suppressed carbohydrate metabolism during desiccation and increased lipid mobilisation both during desiccation stress and during recovery from desiccation. Both the nutrient targeted by the tracer and the timing of the nutrient mobilisation likely contributes to the ability of *C. capitata* to tolerate desiccating conditions. By contrast, *C. rosa* was less flexible than *C. capitata* in terms of mobilising nutrients in a manner that can be interpreted as offsetting desiccation stress. While encountering desiccating conditions, this desiccation-sensitive species increased mobilisation of carbohydrates but decreased protein mobilisation, and only after desiccation stress was lifted was lipid mobilisation increased. Therefore, our hypothesis that a desiccation-resistant species and desiccation-sensitive species will differentially oxidise nutrients as they counter desiccation stress, and that lipids serve as an important fuel source during desiccation stress is supported by our results. By depleting lipid stores instead of protein stores, an invasive beetle was able to rapidly recover from nutritional stress, which potentially gives it an advantage in colonising new areas (Renault et al., 2002). The more desiccation-resistant species tested here (*C. capitata*) was able to rapidly alter lipid oxidation rates to a larger extent than desiccation-sensitive *C. rosa* did, which explains the difference in stress tolerance between these species. We concede that a portion of the glucose tracer could have been converted into lipids (via *de novo* lipogenesis) or into the carbon skeletons of non-essential amino acids and thus could have enriched the lipid and protein pools, respectively, in the body (McCue, 2011). However, because the lipid and the carbohydrate pools of the body account for very similar percentages of the dry mass (Table 1), and given that we observed different macronutrient oxidation responses among the three tracer groups, we believe that we primarily targeted the carbohydrate pool using the glucose tracer treatment.

Stored nutrients and diet play an important role in the stress response of insects, and specific nutrients have been linked to certain stress responses. Weldon et al. (2016) found that desiccated *C. capitata* lost a larger proportion of their body lipid stores than *C. rosa*, suggesting that flexible lipid oxidation may help survival during desiccation. We also observed reduced lipid stores in both species during desiccation; however, interpreting these results is complicated by the use of nutrient tracer-enriched diets, which could affect the body composition of these species. Insects have different quantities of stored nutrients – protein stores can be 45–75% of dry mass (Verkerk et al., 2007) – and these nutrient stores are often affected by diet (Weldon et al., 2019). In *Drosophila*, body lipid and glycogen stores have been linked to both starvation and desiccation resistance (Renault et al., 2002; Gibbs et al., 1997). In some cases, larval diets are more important than adult feeding, as seen in *Ceratitis cosyra* reared on low protein diets, which led to increased desiccation and starvation resistance, despite having lower nutrient stores than flies reared on a standard protein diet (Weldon et al., 2019). Meanwhile, in the same study, stress resistance diminished with adult feeding, regardless of diet nutrient content (Weldon et al., 2019). Stress resistance mechanisms can also be traced to specific nutrients as carbohydrate-rich diets elevate levels of Na^+^/K^+^-ATPase ion transport, which assists in restoring ion balance during recovery from desiccation stress (Sisodia et al., 2015). Similarly, the observed upregulation of carbohydrate mobilisation in *C. capitata* during desiccation stress observed in this study would also indicate that carbohydrates contribute to the desiccation response in this model invasive species.

Multiple stressors often interact with each other, as evidenced by nutritionally and desiccation stressed *C. capitata* having a reduced capacity to deal with thermal stress (Mitchell et al., 2017). Therefore, as stress responses can be energy intensive, there are often trade-offs with other essential functions such as reproduction. Nutrition can also offset these trade-offs as dietary enrichment of carbohydrate and protein increases expression of Hsp60, which plays a vital role in egg production (Sisodia et al., 2015). The rate of nutrient mobilisation is affected by both environmental cues and previous exposure to stress (Williams et al., 2016). *Drosophila* exposed to thermal stress were able to upregulate carbohydrate and protein oxidation, and oxidation rates reduced to previous levels when stress was lifted (Williams et al., 2016). We were able to observe similar patterns of flexible nutrient oxidation in this study, with the more desiccation-tolerant species having a more flexible response.

Global climate change threatens food production by reducing crop yields and altering the nutrient content of staple crops (Flavelle, 2019; Klare, 2007). Moreover, evapotranspiration is likely to increase in the future with climate change (Zhao et al., 2022) and substantially reduce plant moisture content and microsite moisture availability of insects. Thus, the population dynamics of pest insect species will also be affected by changing climates, both directly through exposure to novel and potentially stressful environmental conditions (Duffy et al., 2022; Lehmann et al., 2020) and indirectly by changing diets. Understanding how a pest species will respond to changes in environmental conditions as well as changes in macronutrient availability will enable better predictions of population dynamics as well as assist in developing risk or control strategies. By identifying specific nutrients that affect desiccation resistance in these fruit fly species, we will be able to better predict the spread and persistence of certain pest species under specific climates. Our findings provide clear evidence that lipids play an important part in the desiccation physiology of the desiccation-resistant *C. capitata*. This pattern may extend to other species of ecological or economic importance, including a variety of pollinators and insects mass-reared for genetic control approaches (e.g. Weldon et al., 2019). However, comparisons between only two species are insufficient to draw broader conclusions about the evolution of desiccation adaptations (Goymann and Schwabl, 2021). Future research should therefore expand this framework to include a wider range of species. In addition, studies should quantify ¹³C enrichment in each body pool to ensure that isotopic spillover is properly accounted for and to improve the interpretability and comparability of results.

## Supplementary Material

10.1242/jexbio.250861_sup1 Supplementary information
