# Supplementary material for: Flexible nutrient oxidation during desiccation is associated with climate stress resistance of an invasive fly
Source: J Exp Biol. 2026 Jul 8;229(13):jeb250861. doi: 10.1242/jeb.250861 (PMC13380977; doi:10.1242/jeb.250861)
Supplement: Supplementary information [file jexbio-229-250861-s1.pdf]

Supplementary Materials and Methods

S1 Fly rearing and handling.

Fruit fly colonies for both *C. capitata* and *C. rosa* were established from fruits collected in the Nelspruit region of South Africa. Adult fruit flies were housed in 30 × 30 × 30 cm insect cages (BugDorm, BD6M610) with mesh sleeves for easy handling. Food was provided to adults ad libitum as a 1:1 mixture of granulated sucrose and hydrolysed yeast, and water was provided through damp cotton wool. Eggs were collected from each colony of flies using standard oviposition cups which were constructed from 125 ml disposable plastic cups. In each oviposition cup, a piece of damp tissue paper was placed, and 2 ml of fruit concentrate was evenly distributed over the tissue paper. The oviposition cup was covered with parafilm that was then punctured ~25 times with an insect pin placed inside the sleeves of the bugdroms and fastened with elastic bands so that the parafilm surface was secured in a vertical position. After two days all the eggs were washed from the oviposition cups and transferred to larval diets. A 2 g.L^{-1} solution was prepared for each ^{13}C-tracer, namely ^{13}C-Leucine, ^{13}C-Glucose, and ^{13}C-Palmitic acid, by adding 2 g of dried tracer powder to 1 L of distilled water. A standard larval diet was obtained from Citrus Research International (containing sucrose, desiccated carrot and hydrolysed yeast;8%) and three different enriched larval diets (^{13}C-Leucine, ^{13}C-Glucose, and ^{13}C-Palmitic acid) were prepared by mixing 125 ml of dried powder with 200 ml of the prepared tracer solutions. For each *C. capitata* and *C. rosa,* 3-5 lines of flies were set up by transferring 300 eggs to an enriched larval diet for both the F1 and F2 generations. Individuals from the F2 generation were used in subsequent measurements.

S2 Respirometry calibration and handling

A CO_2/H_2O gas analyser (LiCor 7000, LI-COR Inc., Lincoln, NE, USA) was calibrated using a CO_2 standard (Afrox, South Africa, 350 ppm, balance nitrogen) and a vapour pressure generator for H_2O calibration at 5 °C dew point (LiCor 610, LI-COR Inc., Lincoln, NE, USA). Calibrating the water channel is required to accurately calibrate the CO_2 channel. All water fluctuations in measurements were discarded since it cannot be accurately measured in a multiplexer set up owing to time lags introduced inside the multiplexer. Water loss by each species under desiccating

Journal of Experimental Biology • Supplementary information

conditions were determined in a separate experiment (Bosua et al. 2022). Data were captured using LiCor software (LI-7000 Windows Software Version 2.0.0) to a standard desktop computer at 1 Hz.

Flies were weighed in 2 ml microcentrifuge tubes of predetermined weight. Respirometry was programmed to complete one cycle of all the metabolic chambers sequentially every 2 hours The respirometry chambers were 20 ml plastic syringes that were modified to attach to the flow through respirometry system, with the plunger pushed in to the 5 ml mark.

The test temperature (25 ˚C) was achieved by placing the respirometry chambers containing the individual flies inside a programmable, fluid-filled bath (CC410wl, Huber, Berching, Germany). A thermocouple was inserted into a small opening on the side of the respirometry chamber to monitor the internal temperature for the duration of the metabolic measurement. The opening was sealed after the thermocouple was inserted to keep the chamber gastight. Airflow was controlled at 100 $ml.min^{-1}$ with a mass flow control valve (Sidetrak, Sierra International, USA) coupled to a mass flow control box (Sable Systems International, Las Vegas, NV, USA). Air was first passed through soda lime, silica gel and Drierite columns, to ensure that air that entering the respirometry chamber had 0 % RH and 0 % CO_2. Activity was determined for a single fly per multiplexed run, by recording a voltage spike every time the fly interrupted a light beam, using an electronic activity detector (AD-2, Sable Systems, USA) with data logged to the Licor recording file as an auxiliary channel.

S3 Body composition

A subset of individuals from each species, tracer enrichment, and timepoint were selected for analysis of body water, protein, lipid, and carbohydrate body stores.

Body mass was determined by weighing flies on a Mettler UMX2 ultramicrobalance scale to ±0.0001 mg (CH-8606 Greifensee, Mettler-Toledo GmbH, Laboratory & Weighing Technologies, Switzerland), and subsequently body water was determined by freeze-drying the samples at -80 °C for 48 hours after which they were reweighed to obtain the dry mass weighed again (dry mass), and their body water was determined gravimetrically from the difference between the wet mass and the dry mass.

Body lipids were determined by soaking individual freeze-dried flies in 1 mL 1:12 chloroform: methanol in 1.5 mL microcentrifuge tubes for 24 hours. After 24 hours half of the supernatant was placed into 1.5 mL microcentrifuge tubes and frozen at -80 °C for colorimetric analysis. For the colorimetric analysis, the sulfo-phospho-vanillin method was used in which 50 µl of the solvent was added in triplicate to individual microcentrifuge tubes (1.5 mL). They were then heated at 100 °C for 10 mins to evaporate samples, after which 10 µl of concentrated (> 95 %) sulphuric acid was added to each sample.

Samples with body water and lipids removed were then used to determine proteins in the fly body following Bradford (1976) and Foray et al. (1985). Each sample was placed inside a 1.5 mL microcentrifuge along with a glass bead (3-7 mm diameter) tube, and then submerged in 1 ml of NaOH (0.1 M) and the tubes were and shaken/lysed at room temperature (30 Hz or highest setting, for up to 8 min in 2 min intervals). After this, the tubes were placed on a thermoshaker at 80 °C and 250 RPM for 30 min and subsequently left at room temperature (~25 °C) overnight (~16 h). The following day, the samples were centrifuged for 10 min at 13,000 RPM. After this, 600 µl of supernatant were placed into a separate 1.5 ml tube for protein analysis. For protein analysis, standards were prepared using a PBS buffer (prepared following instructions) and a 4 mg/ml stock solution (40 mg BSA + 10 mL PBS buffer) to achieve the following range of concentrations: (in 0, 0.5, 1.0, 2.0 and 4.0 $mg.ml^{-1}$ standard concentration). Protein concentration of each sample was then determined by adding transferring 1.5 µl of each standard and each sample in triplicate to wells using a 96 well borosilicate microplates (730.009QG, Hellma Analytics, Germany). Following this, 250 µl of Bradford reagent (B6916, Sigma) was added to each sample and the plates were incubated for 15-20 min at room temperature before absorbance was read at 595nm (Tecan Microspark plate reader) and at 25 °C.

Body carbohydrates were determined via the anthrone method from the same supernatant of the reconstituted, freeze-dried, and lipid free samples as were used to determine protein content. A standard curve was drawn up from a glucose dilution range (0.1, 0.3, 0.5, 1.0, 2.5 and 5 $mg.ml^{-1}$). From each sample (standard as well as treatment samples) 200 µl were pipette into 15 ml falcon tubes and 4.8 ml of anthrone reagent (1.42 $g.L^{-1}$ anthrone dissolved in 70 % sulphuric acid) was added. The samples were incubated in a water bath at 90 ºC for 15 min, after which 250 µl from each sample be transferred in duplicate to a microplate reader, and the absorbance of the samples was measured at 625 nm using the microplate reader.

S4 Body mass comparison

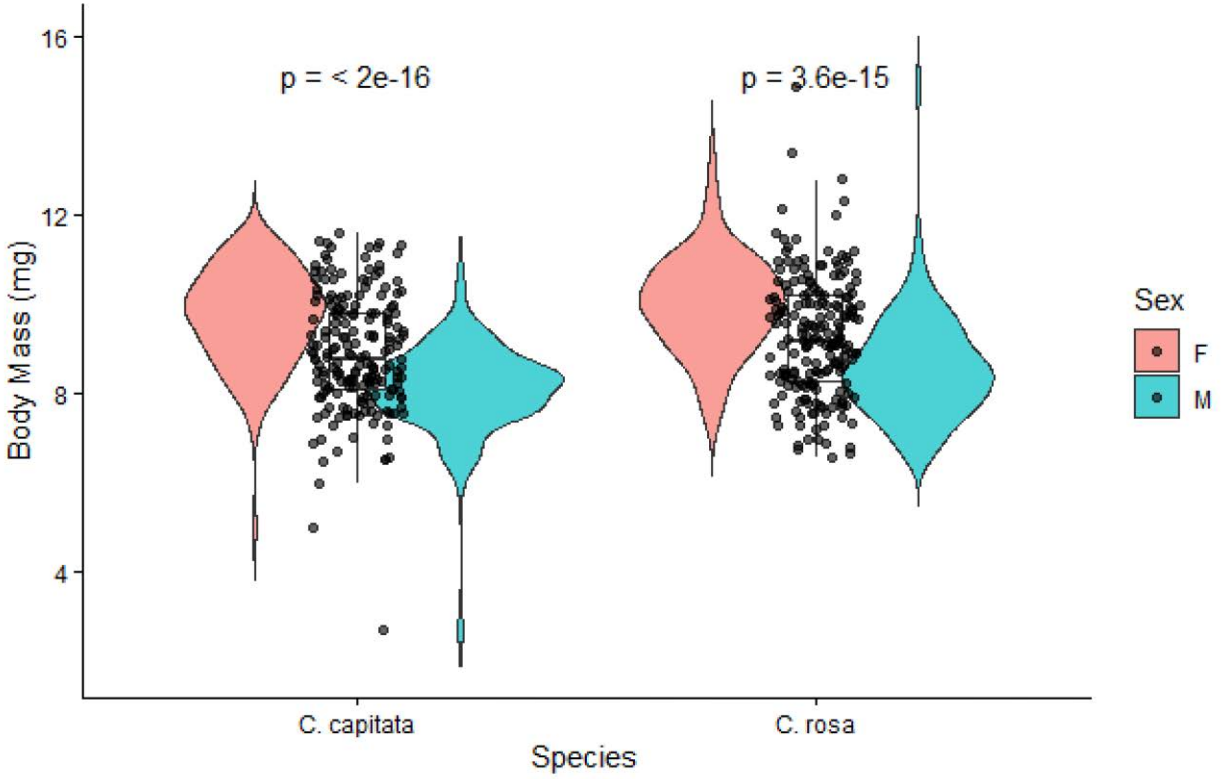

Fig. S1. Body mass comparison between species

Journal of Experimental Biology • Supplementary information
